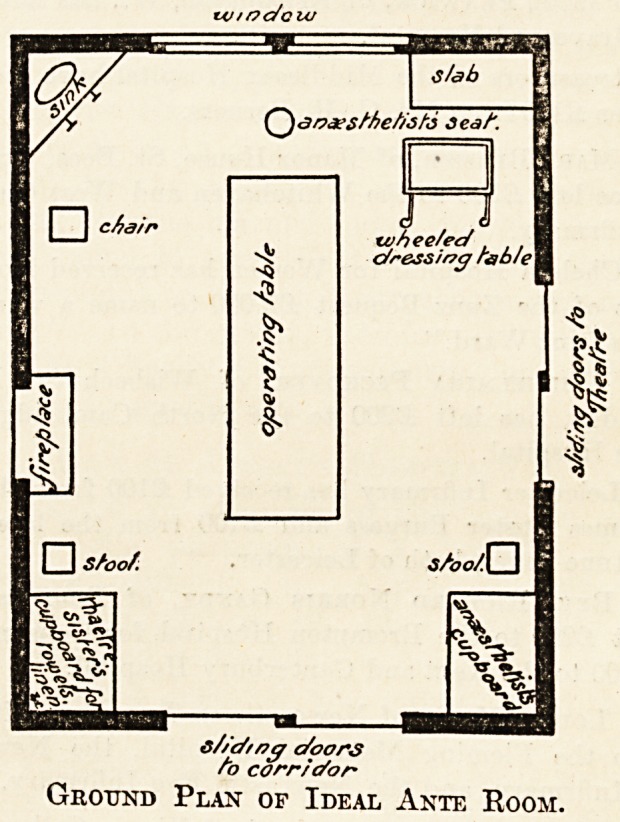# The Anæsthetist's Ante-Room

**Published:** 1910-04-30

**Authors:** 


### Points in Hospital Construction.

### THE ANÆSTHETIST'S ANTE-ROOM.

Even in the most recent and improved London operating theatres, in which lavish expenditure of money and forethought has provided the surgeons with the nearest possible approach to present ideals of surgical perfection, it is too often the case that the anæsthetising room presents numberless points for criticism. When plans for a new theatre are under discussion every surgeon on the staff is taken into consultation with the architect, and every fad is humoured to the uttermost; but to ask an anæsthetist's opinion on the construction or arrangement of the *salle d'anæsthésie* never seems to enter anyone's head. Yet defects so gross and so obvious as to be serious blemishes on the planning of the theatre unit are readily to be found in the anæsthetising rooms of hospitals which pride themselves on their operative facilities. To mention even the serious offenders by name would involve the condemnation of so many English hospitals that it may be more fitting and instructive merely to dwell upon the characteristics of this important though neglected part of theatre designing. All the points mentioned can easily be seen in London alone by anyone who visits a few typical hospitals.

### The Ideal Induction Room.

The anæsthetic room should not be too big, for a large concourse of people is not to be encouraged during the induction of anæsthesia. Where a medical school exists in connection with the hospital teachers should never allow more than two students to be present at a time when a patient is being anæsthetised. Few theatres offend in this way, it is true; but, on the other hand, the ante-room must be large enough, and only too often the anæthetist is unduly cramped for space. It is better that doors should either slide or at any rate not have the ordinary knob-handle of the usual type; the object of this is to avoid noise when the room is entered by somebody during the early stage of induction. The window should be opposite the door, so that the light enters over the anæsthetist's shoulder and is not in the patient's eyes; the latter's feet, when he is disposed on the operating table, thus point to the door, and so he will be wheeled out feet first into the operating theatre. In some ante-rooms there is but one door, giving on to a lobby in which is the door of the theatre. Provided the distance from one door to the other be only a few feet, and provided the plan of preparing the patient in the ante-room be not followed, there is little objection to this; but it is more usual and better to have a separate door communicating directly with the theatre, through which the patient is wheeled when the anæsthetist is ready. A small fireplace should be placed midway between the window and the entrance of the ante-room, opposite the door into the theatre, if there is one. When this arrangement is followed it is impossible for the table to be against the wall during induction, a plan to be avoided, because it gives a struggling patient a leverage for the otherwise somewhat wasted and incoordinate muscular efforts then displayed. If the arms or legs when tossed about come into contact with anything firm and unyielding, as a wall, kicks and struggles become more purposive and more difficult to control.

### The Fittings.

The remaining space at disposal may be usefully allotted somewhat as follows: There should be two cupboards, one for anæsthetic apparatus, such as ether and chloroform, masks, bags, drop bottles, gags, props, and similar implements, and this should be away from the fire, as several of the articles mentioned are either perishable or inflammable; the other should be for porringers, towels, lint, sponges, and other linen such as the theatre sister keeps at the disposal of the anæsthetist. In another corner may be placed a sink with hot and cold water, for the anæsthetist to wash his hands and also his apparatus in. A slab which can be let down when not in use is sometimes put in; it is not essential, but if desired can occupy the remaining corner, handy to the anæsthetist during the induction stage. Better than

Ground Plan of Ideal Ante Room.

this is a railed dressing table on wheels; upon it gags, wedges, tongue forceps, and all the rest of the armamentarium may be disposed, and it can be wheeled straight into the theatre as soon as the patient is transferred thither. Upon the slab, if a wheeled table is provided, may be kept the book in which details of every administration should be entered by the anæsthetist personally; it should be no part of the theatre sister's duties to make entries in this record. Pegs for the coats of the anæsthetists may be provided; and mackintoshes as well as long white overalls should form part of the contents of the linen cupboard. A looking-glass is a useful adjunct, especially in summer, when ties and collars need removal before commencing the day's work, and have subsequently to be readjusted. A clothes brush and a hair brush are equally valuable.

Some Points in Conclusion.

With the outfit of the instrument cupboard it is hardly useful to deal; opinions differ widely as to the best patterns of apparatus, and the tastes of individual administrators are rightly to be consulted. This brief survey may be concluded with some features in construction which should at all hazards be eschewed, though they frequently are not. In no circumstances should there be any door leading from the anæsthetic chamber into either the sterilising room or the surgeon's ante-room. Such an arrangement is not unusual, but it leads to noise and clatter as nurses, students, or surgeons pass across the room to reach the theatre or other parts of the unit. To allow the anæsthetic room to be a public highway, as it only too often is, is a grave fault in theatre architecture. Open fireplaces are better than electric stoves, radiators, or similar devices, because the ventilation of these rooms without causing draughts is always a difficulty. No surgical instruments should be kept in the anæsthetic ante-room, and even the anæsthetist's cupboard should be of wood, not of glass; otherwise a nervous patient may be seriously frightened by the formidable apparatus displayed. A top light, such as is so useful in the operating theatre itself, is quite unnecessary and disadvantageous in the ante-room. It is a mistake to have a special couch for the anæsthetising room, distinct from the operating table, for this involves lifting the patient on to the latter in the theatre. The better plan is to have at least two operating tables for every theatre, one of which is always in the ante-room and one in the theatre itself. They should be similar in design and execution, so that an anæsthetist who is not constantly using them may not fall into any confusion about their working, and mistake one handle or lever or wheel for another. Furniture should be as simple as possible; a couple of stools and a rush-bottom chair, all painted white, are ample. Artificial light should be electric; in fact, no naked light of any sort (other than that of the fire) should be allowed in the room. Smoking by students or anyone else should be strictly prohibited.

## Figures and Tables

**Figure f1:**